# Morphological and molecular divergence of *Rhipicephalus turanicus* tick from Albania and China

**DOI:** 10.1007/s10493-017-0189-8

**Published:** 2017-11-27

**Authors:** Hong-Yu Li, Shan-Shan Zhao, Sándor Hornok, Róbert Farkas, Li-Ping Guo, Chuang-Fu Chen, Ren-Fu Shao, Ji-Zhou Lv, Yuan-Zhi Wang

**Affiliations:** 1 School of Medicine, Shihezi University, Shihezi, 832002 Xinjiang Uygur Autonomous Region People’s Republic of China; 2 Department of Parasitology and Zoology, University of Veterinary Medicine, Budapest, Hungary; 3 College of Animal Science and Technology, Shihezi University, Shihezi, 832002 Xinjiang Uygur Autonomous Region People’s Republic of China; 4 Gene Cology Research Centre, Faculty of Science, Health, Education and Engineering, University of the Sunshine Coast, Maroochydore DC, QLD 4556 Australia; 5 Institute of Animal Quarantine, Chinese Academy of Inspection and Quarantine, Beijing, 100029 People’s Republic of China

**Keywords:** *Rhipicephalus turanicus*, Subspecies, Morphology, Phylogeny

## Abstract

*Rhipicephalus turanicus* is an important tick species potentially carrying tick-borne pathogens. Several tick species have obvious subspecies divergence. However few studies aimed to examine the existence of divergence within *R. turanicus*. Therefore, a detailed morphological and molecular analysis was conducted for comparing *R. turanicus* from the Mediterranean Basin (represented by Albania) and Central Asia (Northwestern China). Altogether 315 adult ticks of *R. turanicus* (103 from Albania and 212 from China) were morphologically and molecularly analysed. DNA samples were used for mitochondrial *16S rRNA* and *cox1* gene sequences analysis. In addition, as potentially genetic markers, three fragments including partial *nad1*-*16S rRNA*, *nad2*-*cox1*, *cox1*-*tRNA*-*Lys*, were designed and then phylogenetically analyzed. Based on detailed morphological observations, only basis capituli length:width ratio (females), the length, the width and the length:width ratio of the scutum (males) had differences between *R. turanicus* from China and Albania. Gene divergences of *16S rRNA*, *cox1*, partial *nad1*-*16S rRNA*, *nad2*-*cox1* and *cox1*-*tRNA*-*Lys* from China and Albania ticks were 3.53–4.84, 3.57–4.92, 3.57–4.07, 3.57–4.39 and 3.18–4.69%, respectively. The evaluated five genetic markers revealed two phylogenetic branches in *R. turanicus*. Obvious differences exist within *R. turanicus* based on morphological and genetic analysis. Three newly designed genetic markers (partial *nad1*-*16S rRNA*, *nad2*-*cox1* and *cox1*-*tRNA*-*Lys*) in this study may be suitable genetic tools for identification and analysis in *R. turanicus*. Subspecies analysis of *R. turanicus* from other regions of the world should be initiated in the future.

**Electronic supplementary material:**

The online version of this article (10.1007/s10493-017-0189-8) contains supplementary material, which is available to authorized users.

## Introduction

*Rhipicephalus turanicus* is widely distributed in the Mediterranean sub-region, Africa and Asia, infesting a rich variety of domestic and wild hosts such as sheep, cattle, horses, dogs, cats, and Corsican hares, occasionally even human beings (Chochlakis et al. 2014; Dantas-Torres et al. 2013). It is a potential or competent vector of several categories of tick-borne pathogens such as *Rickettsia* spp. (Germanakis et al. 2013) among various hosts and negatively impacts agricultural economies globally. While several tick species have valid subspecies around the world, as exemplified by *R. evertsi* (Guglielmone et al. 2014) and *Haemaphysalis erinacei* (Hornok et al. 2016), few studies aimed to examine the existence of distinction within *R. turanicus*, despite the known uncertainty in its taxonomy (Dantas-Torres et al. 2013).

*Cox1* and *16S rRNA* genes are well established barcoding genes for the molecular identification and phylogenetic analyses of ticks. Intraspecies sequence divergence within *R. turanicus* from eastern and central parts of China has been investigated (Lv et al. 2014). Intraspecies genetic variation in the *16S rRNA* and cytochrome c oxidase subunit I (*cox1*) target region have been recorded concerning *R. turanicus* in Yining County, Xinjiang Uygur Autonomous Region (XUAR) (Du et al. 2015), where *R. turanicus* was considered one of the dominant tick species (Wang et al. 2015).

Here, based on complete mitochondrial sequence analysis of the members of *Rhipicephalus sanguineus* complex (with at least 17 species), 3 mitochondrial hypervariable region fragments, namely, partial *nad1*-*16S rRNA* gene, partial *nad2*-*cox1* gene and partial *cox1*-*tRNA*-*Lys* gene, together with *Cox1* and *16S rRNA* fragments and detailed morphological study were analysed to investigate divergence of *R. turanicus* originated from Albania and Northwestern China.

## Material and methods

### Tick collection

Two hundred and twelve *R. turanicus* (infesting sheep) were collected from seven counties/cities, including Yining, Fukang, Alataw, Pishan, Qira, Yecheng, and Tumxuk. Similarly, one hundred and three *R. turanicus* from Albania (originated from Helmes-Kavaj, Merrge Lezhe, Lakaret Gjirokaster and Librakhol-Goravash regions) were also used for study. Collected locations and partial submitted sequences information see (Additional Table 1).

### Morphological analysis

Ticks were first identified as *R. turanicus* according to their morphological characteristics based on the work of Walker et al. (2000) and Filippova (1997). Pictures were made and measurements were performed with a Keyence VHX-5000 digital microscope (Osaka, Japan). The same parameters were measured and compared in the case of males (92 from China, 53 from Albania) and females (120 from China, 50 from Albania), except for the peritreme process (used only for females). Morphological study included anteriolateral setae (palp article II), anterior setae (palpal article III), inner length (palpal articles II + III), outer length (palpal articles II + III), palpal width (between palpal articles II/III), basis capituli length, basis capituli width, ratio basis capituli length:width, scutum median length, scutum width, ratio scutum length:width, peritreme median length, peritreme width, peritreme process width and peritreme process median length. Structures of females measured are illustrated in Additional Figure 1. Measurements of ticks were compared with Student *t* test. Mean values were considered significantly different if *P* < 0.05 (Additional Table 3).

### Molecular analysis

Total genomic DNA of 315 *R. turanicus* (212 from China and 103 from Albania) was extracted from individual specimens using the TIANamp Genomic DNA Kit (TIANGEN, Beijing, China).

All ticks were first evaluated based on *16S rDNA* and *cox1* genetic fragments (Black and Piesman 1994; Chen et al. 2014). Moreover, 13 complete mitochondrial genomes of *Rhipicephalus* spp. available in GenBank were used to analyze their conservative motifs by DNAMAN 6.0 software (Additional Table 2). Three pairs of primers aimed to hypervariable region fragments were designed by Primer Premier 5.0 software, and their cycling conditions were shown in Additional File 1. The 654 bp fragment of *N1* (partial *nad1*-16S rRNA gene), 595 bp fragment of *N2* (partial *nad2*-*cox1* gene), and 780 bp fragment of *C1* (partial *cox1*-*tRNA*-*Lys* gene) were used as novel genetic markers to analyze divergence of *R. turanicus*. Data analysis methods are described in references (Dantas-Torres et al. 2013; Lv et al. 2014). PCR products were purified using the TIANgel Midi Purification Kit (NGEN, Beijing, China), and sequenced by Sangon Biotech (Shanghai, China). The automatic MEGA model selection method (analysis: Maximum Likelihood model selection, substitution type: nucleotide) was applied to choose the appropriate model for phylogenetic analyses. The dataset was resampled 1000 times to generate bootstrap values. Phylogenetic analyses were conducted with the Maximum Likelihood method by using MEGA v.6.0. Outgroups of phylogenetic trees were selected from GenBank. The 75 nucleotide sequences (*16S rDNA*: KY583065-KY583081; *cox1*: KY606287-KY606303; *N1*: KY620098-KY620114; *N2*: KY626023-KY626039; and *C1*: KY996824-KY996840) were deposited in the GenBank database.

## Results and discussion

Concerning evaluated morphological parameters, only the ratio basis capituli length:width was significantly different between *R. turanicus* females from China and Albania (*P* = 0.022). Three parameters were significantly different between *R. turanicus* males from China and Albania, i.e. the length, the width and the length: width ratio of the scutum (*P* = 0.0125, 0.0018 and 0.0027, respectively) (Additional Table 3). The observed differences between one parameter of female and three parameters of male *R. turanicus* collected in China and Albania might be partialy interpreted as geographically related intraspecific morphological variations. Similar differences were noted by Filippova (1997) when comparing *R. turanicus* from four geographical regions in Central Asia (Dagestan, Badaj-Tugaj, Taskent, Sumbar).

**Fig. 1:**
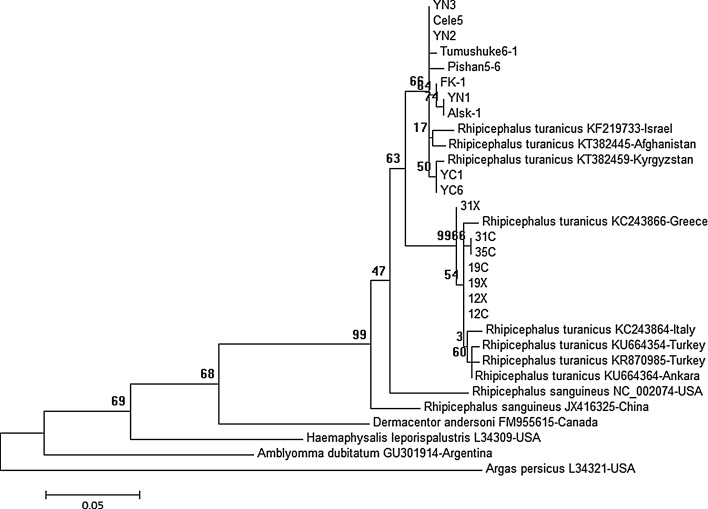
Phylogenetic comparison of *16S rDNA* sequences of *Rhipicephalus turanicus*. The genotypes of ticks from this study are marked with location and isolate code. Branch lengths represent the number of substitutions per site inferred according to the scale shown

**Fig. 2:**
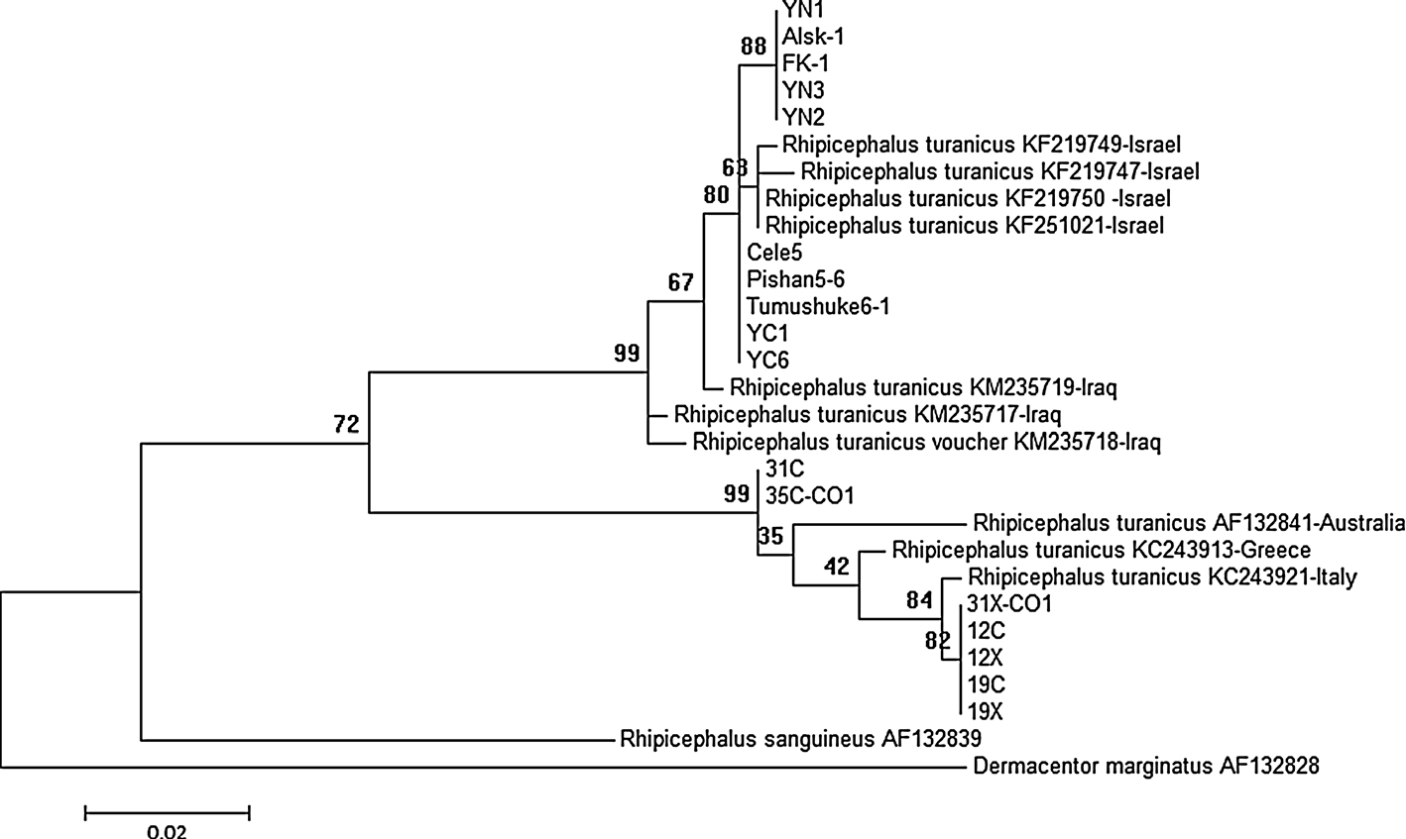
Phylogenetic relationships of *Rhipicephalus turanicus*, based on the amplified part of the *cox1* gene. The genotypes of ticks from this study are marked with location and isolate code. Branch lengths represent the number of substitutions per site inferred according to the scale shown

Sequencing data confirmed the morphological results on the basis of BLASTn analysis of *16S rRNA* and *cox1* gene, as these had 98.83–99.56 and 98.71–99.88% homology with the GenBank Italian and American sequences of *R. turanicus*. Based on *16S rDNA* sequence data and Italian *R. turanicus* reference sequence (accession number: KC243864) (Dantas-Torres et al. 2013), 315 *R. turanicus* ticks indicated two different lineages of *R. turanicus* originated from from northwestern China and Albania, respectively (Fig. 1). The analysis of sequence divergences showed that the *16S rRNA* sequences from Chinese *R. turanicus* were 0–1.32% whereas from Albania they were 0–0.44%. Interestingly, the sequence divergences between *R. turanicus* from Albania and China were 3.53–4.84%. In addition, based on *cox1* sequence data, two significantly different lineages also appeared in *R. turanicus* ticks (Fig. 2). The analysis of sequence divergences showed that the *cox1* sequences from Chinese inners, Albania inners and Chinese-Albania interface were 0–0.47, 0–1.42 and 3.57–4.92%, respectively. The latter value might inspire us whether subspecies within it or species complex (Dantas-Torres et al. 2013). *16S rRNA* gene phylogenetic trees showed two branches were presented in *R. turanicus*. All *R. turanicus* sequences of northwestern China clustered into the same group, which contains *16S rRNA* gene sequences of Kyrgyzstan (KT382459), Israel (KF219733), and Afghanistan (KT382445), whereas all sequences from Albania clustered into another group combined with sequences from Italy reference sequence (KC243864) (Dantas-Torres et al. 2013), Greece (KC242866), and Turkey (KU664364 and KU664354) (Fig. 1). Similarly, based on *cox1* phylogenetic analysis, *R. turanicus* sequences of northwestern China were in the same phylogenetic group, which included sequences from Israel (KF219719, KF219747, KF219750 and KF251021) and Iraq (KM235717-KM235719). Meanwhile, together with KC243921 (from Italy reference sequence) (Dantas-Torres et al. 2013), KC243913 (from Greece) and AF132841 (from Australia), the sequences originating from Albania clustered into another branch (Fig. 2).

**Fig. 3:**
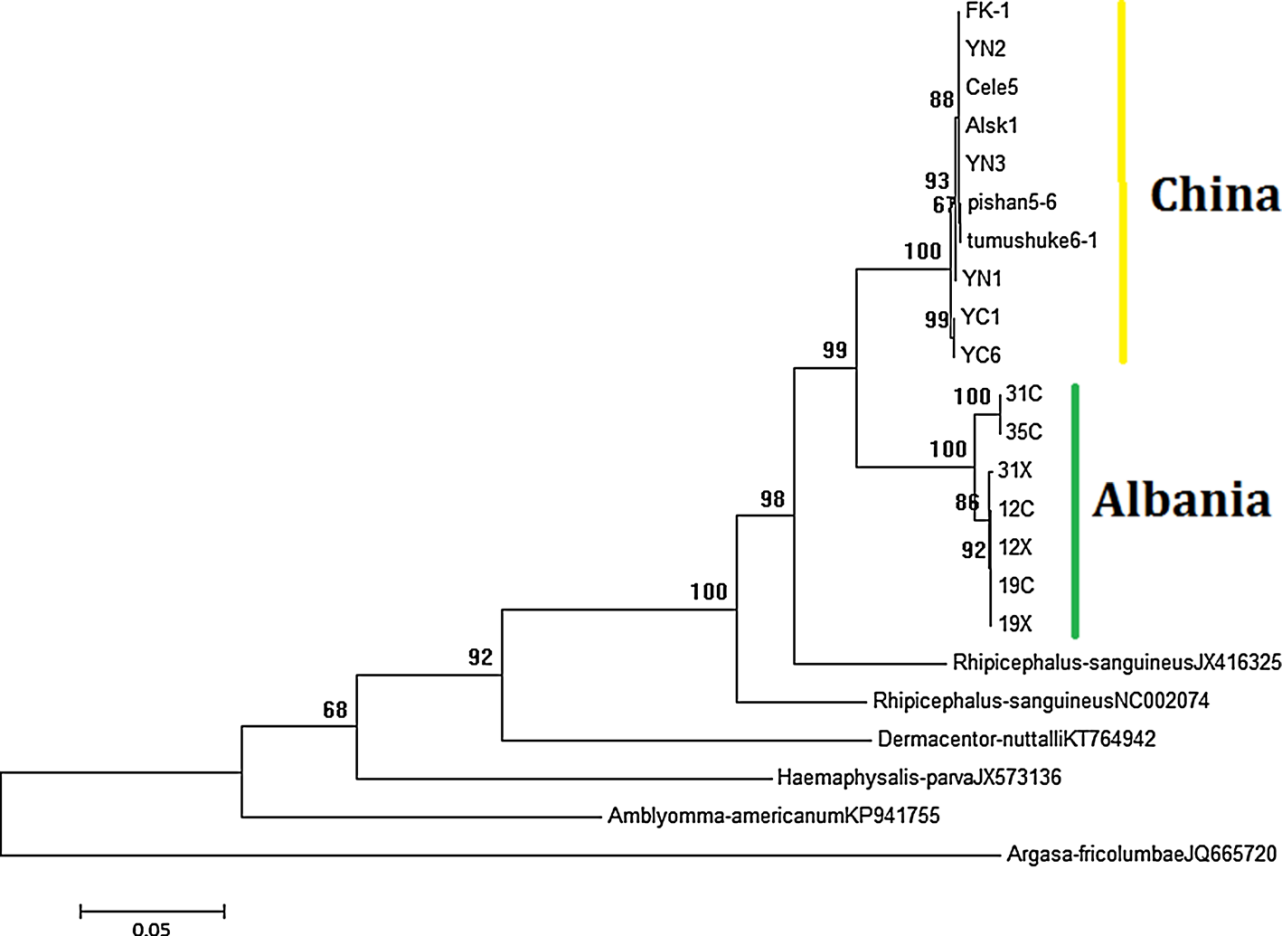
Phylogenetic tree of the partial *N1*-*N2*-*C1* concatenated sequence of *Rhipicephalus turanicus*. The genotypes of ticks from this study are marked with location and isolate code. Branch lengths represent the number of substitutions per site inferred according to the scale shown. The vertical yellow and green lines mark the *R. turanicus*, originated from China and Albania respectively. (Color figure online)

Three pairs of newly primers aimed to hypervariable mitochondrial region fragments were designed based on 13 complete mitochondrial genomes of *Rhipicephalus* spp. available in GenBank, which are superior to *16S rRNA* and *cox1* gene owing to their widely coverage than *16S rRNA* and *cox1* gene. When analysing three novel genetic markers (*N1*, *N2* and *C1*), two different lineages also appeared respectively in 315 *R. turanicus* ticks (Fig. 3). The sequence divergences showed that the *N1*, *N2* and *C1* sequences from Chinese inners were 0–0.46, 0–1.01 and 0–0.87%, respectively. The divergences from Albania inners were 0–1.22, 0–1.27 and 0–1.67%, while the data from Chinese-Albania interface was 3.57–4.07, 3.57–4.39 and 3.18–4.69%, respectively. Our three genetic markers of concatenated phylogenetic analysis also support two branches in *R. turanicus*, thus these three genetic markers may be suitable genetic tools for distinction identification analyses (Fig. 3). More ticks originated from other part of the world should be molecularly analyzed by 3 newly markers for the divergence of *R. turanicus*.

In this study, *R. turanicus* originated from northwestern China were collected in semi-desert regions, neighbored to Takla makan desert or Gurbantunggut desert. Interestingly, as for geographical characteristics, Afghanistan, Kyrgyzstan and Israel belong to semi-desert or desert regions, which may support that *R. turanicus* evolved in association with hosts of similar biotopes, although diversification of this lineage was shown based on phylogenetic analysis of *16S rDNA*, *cox1* and three newly markers data. On the other hand, Albania, Greece and Italy are situated in the Mediterranean Basin, with warm and humid climate in winter and dry and high-temperature in summer, which might have partially contributed to the evolution of different *R. turanicus* lineage compared to northwestern China-originated ones. Accordingly Filipe Dantas-Torres pointed out the existence of different species or taxonomical units within *R. turanicus* (Dantas-Torres et al. 2013).

Overall, morphological, molecular data divergence and phylogenetic trees based on five mitochondrial genetic markers showed that two genetic branches might exist within *R. turanicus*. Phylogenetic analyses generated trees that segregated our tick sequences together with sequences in GenBank, into two distinct clades: one is represented by Central Asia region; the second clade is from the European region, which is in line with former observations by Dantas-Torres on *R. sanguineus* sensu lato from the New and Old worlds (Dantas-Torres et al. 2013). Herein, our findings suggest that *R. turanicus* samples evaluted here from two geographically distant regions showed distinctly divergence. In the future, more efforts are needed to study this, based on a larger number of *R. turanicus* specimens collected throughout its vast geographical range around the world.

## Conclusion

The present study showed the existence of morphological and molecular divergence among *R. turanicus* (from Albania and China), which needs to be further analysed by larger scale sampling of this species in the Palaearctic. Three novel genetic markers (*N1*, *N2* and *C1*), in addition to *16S rRNA* and *cox1* gene, may be suitable genetic tools of *R. turanicus* phylogenetic analysis for distinction delineation.

## Electronic supplementary material

Below is the link to the electronic supplementary material.

Supplementary material 1 (DOC 44 kb)

Supplementary material 2 (DOC 53 kb)

Supplementary material 3 (DOC 38 kb)

Supplementary material 4 (DOC 43 kb)

Supplementary material 5 (JPEG 506 kb)
