# Supplementary material for: Morphological and molecular divergence of Rhipicephalus turanicus tick from Albania and China
Source: Exp Appl Acarol. 2017 Nov 27;73(3):493–9. doi: 10.1007/s10493-017-0189-8 (PMC5727151; doi:10.1007/s10493-017-0189-8)
Supplement: Supplementary file 1 — Supplementary material 1 (DOC 44 kb) [file 10493_2017_189_MOESM1_ESM.doc]

**Additional File 1**

The PCR equipment was a Techne TC-412 thermal cycler, Barloworld Scientific, Cambridge, UK.

**1. PCR amplification of *16S rDNA* mitochondrial gene sequences from 17 tick specimens**

Each reaction consisted of 1 μL of tick genomic DNA (50 ng) and 12.5 μL of a PCR mix containing 50 mM KCl, 10 mM Tris-HCl (pH 8.3), 1.5 mM MgCl_2_, 250 μM of each dNTP, 40 pmol of each primer (*16S rDNA*), and 1.0 U of Taq DNA polymerase (TaKaRa Taq Version 2.0, Takara, Dalian, China).The cycling conditions consisted of an initial 5-min denaturation at 94°C, followed by 38 cycles at 94°C for 30 s, 54°C for 30 s, and 72°C for 1 min, with a final extension at 72°C for 8 min.

1. **PCR amplification of** ***cox1* gene mitochondrial gene sequences from 17 tick specimens**

Each reaction consisted of 1 μL of tick genomic DNA (50 ng) and 12.5 μL of a PCR mix containing 50 mM KCl, 10 mM Tris-HCl (pH 8.3), 1.5 mM MgCl_2_, 250 μM of each dNTP, 40 pmol of each primer (*cox1* gene), and 1.0 U of Taq DNA polymerase (TaKaRa Taq Version 2.0, Takara, Dalian, China).The cycling conditions consisted of an initial 5-min denaturation at 94°C, followed by 40 cycles at 94°C for 30 s, 45°C for 45 s, and 72°C for 1 min, with a final extension at 72°C for 8 min.

1. **PCR amplification of N1, N2 and C1 gene mitochondrial gene sequences from 17 tick specimens**

Each reaction consisted of 1 μL of tick genomic DNA (50 ng) and 12.5 μL of a PCR mix containing 50 mM KCl, 10 mM Tris-HCl (pH 8.3), 1.5 mM MgCl_2_, 250 μM of each dNTP, 40 pmol of each primer (*cox1* gene), and 1.0 U of Taq DNA polymerase (TaKaRa Taq Version 2.0, Takara, Dalian, China). The cycling conditions consisted of an initial 5-min denaturation at 94°C, followed by 35 cycles at 94°C for 45 s, 50°C for 45 s (N1, C1 primer) and 58°C for 45 s (N2 primer), and 72°C for 80 s, with a final extension at 72°C for 8 min.

| Gene | Primer | Sequence(5’-3’) | Reference |
| --- | --- | --- | --- |
| *16S rDNA* | 16s-F | CTGCTCAATGATTTTTTAAATTGCTGTGG | [[1](#_ENREF_1)] |
|  | 16s-R | CCGGTCTGAACTCAGATCAAGT | [[1](#_ENREF_1)] |
| *cox1* | Cox1-F | AATTTACAGTTTATCGCCT | [[2](#_ENREF_2)] |
|  | Cox1-R | CATACAATAAAGCCTAATA | [[2](#_ENREF_2)] |
| *N1* | N1-F | ATTATGGCTATTCTTACTTC | This study |
|  | N1-R | ATTTCCTACATGATCTGAGT | This study |
| *N2* | N2-F | CCATCAAGGATGAATTTTATGT | This study |
|  | N2-R | GTAGAATATATTCATCGCGG | This study |
| *C1* | C1-F | CCATCAGAACACTCTTTCAA | This study |
|  | C1-R | TGGTTTAAGAGACCATCAC | This study |

**References**

[1] Black WC, Piesman J. Phylogeny of hard and soft-tick taxa (Acari: Ixodida) based on mitochondrial 16 s rDNA sequences. Proc Natl Acad Sci USA. 1994;91:10034-8

[2] Chen Z, Li Y, Ren Q, Luo J, Liu Z, Zhou X, et al. Dermacentor everestianus hirst, 1926 (acari: ixodidae): phylogenetic status inferred from molecular characteristics. Parasitology Research, 2014;113(10): 3773-9.
