# Supplementary material for: Morphological and molecular divergence of Rhipicephalus turanicus tick from Albania and China
Source: Exp Appl Acarol. 2017 Nov 27;73(3):493–9. doi: 10.1007/s10493-017-0189-8 (PMC5727151; doi:10.1007/s10493-017-0189-8)
Supplement: Supplementary file 2 — Supplementary material 2 (DOC 53 kb) [file 10493_2017_189_MOESM2_ESM.doc]

**Additional** **Table 1** Collection location, host and sequence ID of *Rhipicephalus turanicus* submitted sequences

| Tick ID | Country | Location | Host | 16S rDNA sequence ID | *cox1* sequence ID |
| --- | --- | --- | --- | --- | --- |
| YN1 | China | Yining | sheep | KY583065 | KY606287 |
| YN2 | China | Yining | sheep | KY583066 | KY606288 |
| YN3 | China | Yining | sheep | KY583067 | KY606289 |
| FK1 | China | Fukang | sheep | KY583068 | KY606290 |
| Alsk-1 | China | Alataw | sheep | KY583069 | KY606291 |
| YC1 | China | Yecheng | sheep | KY583070 | KY606292 |
| YC6 | China | Yecheng | sheep | KY583071 | KY606293 |
| Tumushuke-1 | China | Tumxuk | sheep | KY583072 | KY606294 |
| Cele-5 | China | Qira | sheep | KY583073 | KY606295 |
| Pishan5-6 | China | Pishan | sheep | KY583074 | KY606296 |
| 12C | Albania | Helmes-Kavaj | goat | KY583075 | KY606297 |
| 12X | Albania | Helmes-Kavaj | goat | KY583076 | KY606298 |
| 19C | Albania | Merrge Lezhe | sheep | KY583077 | KY606299 |
| 19X | Albania | Merrge Lezhe | sheep | KY583078 | KY606300 |
| 31C | Albania | Lakaret Gjirokaster | sheep | KY583079 | KY606301 |
| 31X | Albania | Lakaret Gjirokaster | sheep | KY583080 | KY606302 |
| 35C | Albania | Librakhol (Goravash) | sheep | KY583081 | KY606303 |
