# Supplementary material for: Morphological and molecular divergence of Rhipicephalus turanicus tick from Albania and China
Source: Exp Appl Acarol. 2017 Nov 27;73(3):493–9. doi: 10.1007/s10493-017-0189-8 (PMC5727151; doi:10.1007/s10493-017-0189-8)
Supplement: Supplementary file 3 — Supplementary material 3 (DOC 38 kb) [file 10493_2017_189_MOESM3_ESM.doc]

**Additional** **Table 2.** Thirteen complete mitochondrial sequences of *Rhipicephalus*.

| Name | Sequence ID | Length (bp) |
| --- | --- | --- |
| *R. sanguineus* | JX416325 | 14714 |
| *R. sanguineus* | NC_002074 | 14710 |
| *R.australis* | KC503255 | 14891 |
| *R.australis* | NC_023348 | 14891 |
| *R.geigyi* | KC503263 | 14948 |
| *R.geigyi* | NC_023350 | 14948 |
| *R.microplus* | KC503259 | 14864 |
| *R.microplus* | KC503260 | 14903 |
| *R.microplus* | KC503261 | 14905 |
| *R.microplus* | KJ522808 | 14901 |
| *R.microplus* | KP143546 | 15167 |
| *R.microplus* | NC_023335 | 14905 |
| *R.simus* | KJ739594 | 14929 |
