# Supplementary material for: Morphological and molecular divergence of Rhipicephalus turanicus tick from Albania and China
Source: Exp Appl Acarol. 2017 Nov 27;73(3):493–9. doi: 10.1007/s10493-017-0189-8 (PMC5727151; doi:10.1007/s10493-017-0189-8)
Supplement: Supplementary file 4 — Supplementary material 4 (DOC 43 kb) [file 10493_2017_189_MOESM4_ESM.doc]

**Additional Table 3. Measurements (mean values and standard deviation, expressed in µm) of *R. turanicus* from China and Albania.**Two values in the same row, which are significantly different, are shaded with the same hue of grey.

| **Structure** | **Male** | | **Females** | |
| --- | --- | --- | --- | --- |
|  | **China (n=92)** | **Albania (n=53)** | **China (n=120)** | **Albania (n=50)** |
| **anteriolateral seta (palp article II)** | 37.5±2.2 | 37.2±2.2 | 35.1±2.8 | 31.6±1.6 |
| **anterior seta (palpal article III)** | 22.1±3.7 | 23.6±1.7 | 21.3±1.7 | 20.5±2.7 |
| **inner length (palpal articles II+III)** | 188±14.7 | 192.3±9.4 | 201.8±17.2 | 199.9±15.6 |
| **outer length (palpal article II+III)** | 191.1±7.3 | 180.8±8.5 | 198.5±14.9 | 195.2±12.8 |
| **palpal width (between palpal articles II/III)** | 134.1±17.9 | 130.6±11 | 118.8±7.7 | 116.3±10.5 |
| **basis capituli length** | 268±40.9 | 264.6±9.7 | 204.9±14.1 | 226.8±14.4 |
| **basis capituli width** | 528.4±66.4 | 531.1±24.3 | 517.3±22.4 | 533.1±22.5 |
| **ratio basis capituli length:width** | 0.508±0.015 | 0.498±0.012 | 0.396±0.011 | 0.428±0.021 |
| **scutum median length** | 2931.5±102.4 | 2704.7±113.6 | 1192±146.4 | 1240.5±89.9 |
| **scutum width** | 2021.5±83.6 | 1770.4±85.8 | 1215.2±98.6 | 1318.8±145 |
| **ratio scutum length:width** | 1.45±0.012 | 1.53±0.034 | 0.978±0.04 | 0.943±0.06 |
| **peritreme median length** | 518.2±62.9 | 490.6±20.7 | 349.1±13 | 358.5±36.8 |
| **peritreme width** | 196.6±29.8 | 204.7±14.4 | 214±22.3 | 226.5±13.8 |
| **peritreme process width** | - | - | 111.9±15.3 | 108.9±7.3 |
| **peritreme process median length** | - | - | 77.5±13.7 | 67.5±17.9 |
