# Supplementary figures and images for: Morphological and molecular divergence of Rhipicephalus turanicus tick from Albania and China
Source: Exp Appl Acarol. 2017 Nov 27;73(3):493–9. doi: 10.1007/s10493-017-0189-8 (PMC5727151; doi:10.1007/s10493-017-0189-8)

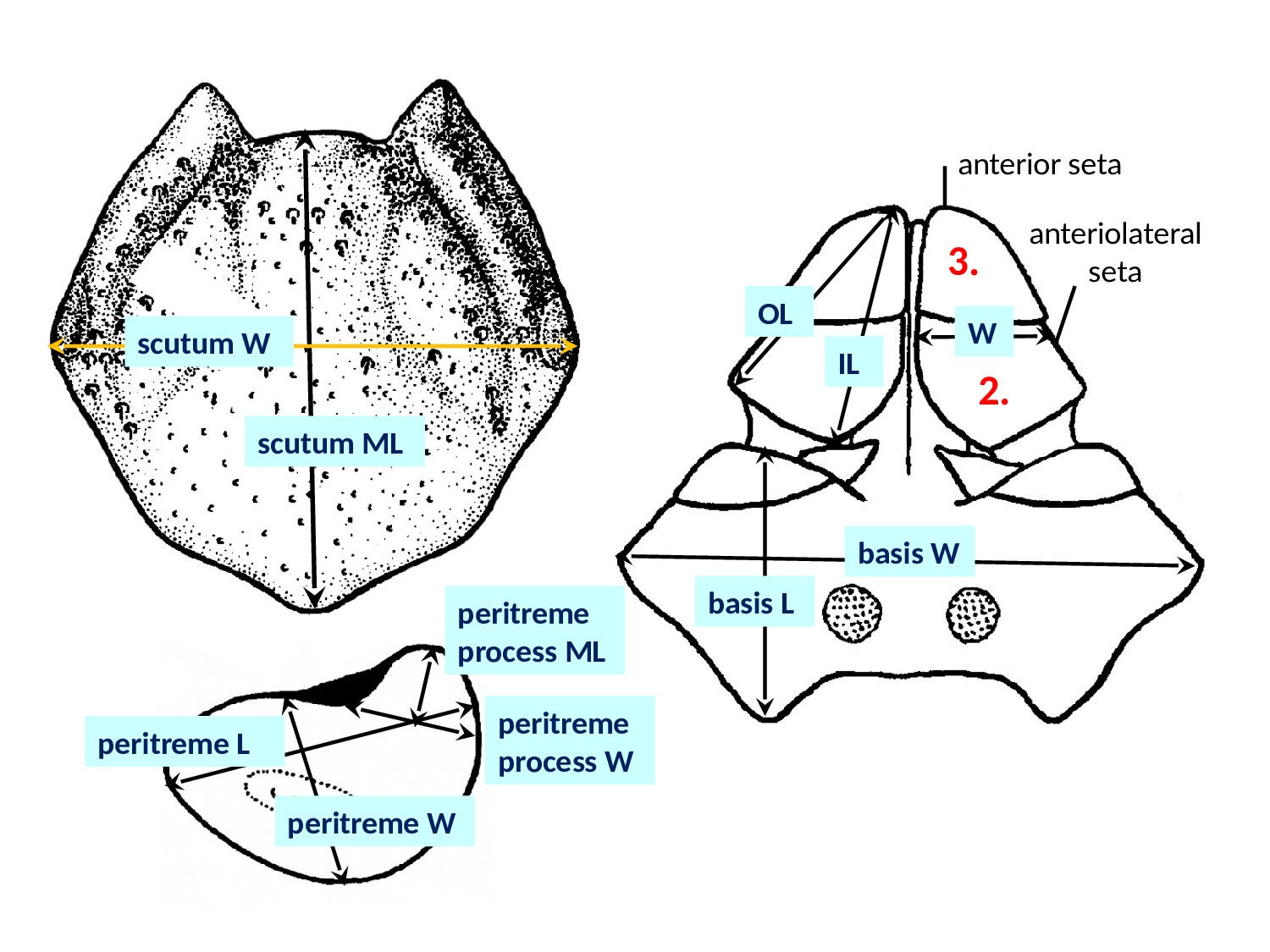

Supplement: Supplementary file 5 — Supplementary material 5 (JPEG 506 kb) [file 10493_2017_189_MOESM5_ESM.jpg]
